# Ethyl Extract of *Blaps rynchopetera* Fairmaire Ameliorates Experimental Aerobic Vaginitis by Inhibiting Autophagy Activation

**DOI:** 10.1155/2022/7938733

**Published:** 2022-02-08

**Authors:** Jia Liu, Siyi Yuan, Zhiming Zhao, Huixia Lu

**Affiliations:** ^1^Department of Gynecology, College of Clinical Medical, Dali University, Dali 671000, China; ^2^Tobacco Company of Dali, Dali 671000, China

## Abstract

To investigate the therapeutic effects of *Blaps rynchopetera* Fairmaire (BRF) extracts on the autophagy activation in a rat model of aerobic vaginitis (AV), forty-eight adult female SD rats of the AV model were divided into five groups according to different treatments with the cream matrix, antibiotics (clindamycin phosphate, 20 mg/g), low-concentration extracts (100 mg/g), medium-concentration extracts (200 mg/g), or high-concentration extracts (300 mg/g). Two weeks after treatment, we conducted a series of assays, including measuring the improved Donders score and pH value of vaginal secretions, H&E staining, immunohistochemical (IHC) staining, western blotting, and ELISA, for evaluating the protein expression of autophagy biomarkers Beclin1 and LC3B. *In vitro,* BRF ethyl extracts had the lowest minimum inhibitory concentration (MIC) and minimum bactericidal concentration (MBC). After administration in AV rats, the improved Donders score, pH value, and vaginal histopathology score of vaginal secretions in the model and low-concentration groups were not statistically different from those in the normal group. In contrast, these indicators improved significantly in other treatment groups, especially in the high-concentration group. Additionally, when compared to the normal group, IHC, western blot, and ELISA revealed no statistically significant difference in the expressions of Beclin1 and LC3B in the model and low-concentration groups, but decreased to varying degrees in the clindamycin, medium-concentration, and high-concentration groups, with no correlation between Beclin1 and LC3B. Medium- and high-concentration BRF ethyl extracts effectively treated AV in rats by inhibiting excessive autophagy activation and could be applied in the clinic future.

## 1. Introduction

Aerobic vaginitis (AV) is a common vaginal infection caused by mono- or multiaerobic germs, characterized by yellow-green purulent leucorrhoea, vulval congestion, swelling, and thermalgia. AV accounts for 5%–23.7% of all vaginitis cases and is associated with poor pregnancy outcomes and even cervical intraepithelial neoplasia (CIN) [1]. AV lacked effective therapeutic options, and the use of long-term antibiotics can cause resistance and irreversible damage to the vaginal microbial evaluation system (VMES). Consequently, exploring novel therapeutic targets has emerged as a frontline area of AV research.

The etiology of AV is unknown, but it may be related to abnormal estrogen levels, immune system disorders, and vitamin D deficiency in women. Autophagy is a type of programmed cell death (PCD) that occurs in all eukaryotic cells. When exposed to hypoxia, starvation, or bacterial infection, autophagy-induced proteins such as Beclin1 and microtubule-associated protein 1 light chain 3 (LC3B) are activated. LC3B and Beclin1 were detected in different stages of autophagy. LC3B is a membrane-specific protein that exists in two forms, LC3B-I and LC3B-II, with the expression level of the LC3B-II protein serving as an indicator for the number of autophagosomes and the degree of autophagy. Beclin1 is an indispensable gene of autophagosome formation. Both LC3B and Beclin1 could be used to evaluate the extent of autophagy.

Earlier studies revealed that bacterial infection in the female reproductive system, such as AV, activates autophagy and alters biomarkers [1]. Regulating the autophagy pathway by targeting relevant biomarkers may become a feasible treatment strategy for AV.

Lately, various chemical substances derived from insects with potent biological activities and fewer side effects have been used in drug discovery. *Blaps rynchopetera* Fairmaire (BRF) is a medicinal insect of the family Tenebrionidae (Coleoptera), found in the Yunnan-Guizhou Plateau of China and widely used to treat fever, bruises, and complex diseases [2, 3]. Its medicinal properties include anti-inflammatory, antioxidation, antitumor, and immunity-boosting [4–6]. Additionally, phenolics of BRF have antibacterial activity *in vitro*, especially against Gram-positive bacteria. However, few reports about their application in bacterial vaginitis exist in the literature [7].

In this study, we evaluated the vaginal treatment effects of various BRF extracts in an AV rat model. We also investigated their effect on the expression levels of the autophagy biomarkers Beclin1 and LC3B. We aimed to generate a novel treatment strategy for AV disease by utilizing BRF extracts.

## 2. Materials and Methods

### 2.1. BRF Extraction

Pro. Yang Zizong of Insect Medicine Biology Research Institute of Dali University has identified 3000 g of live adult BRF as a suitable sample. For extraction, 3000 g of live adult BRF was soaked in 60% ethanol for 1 hour before being mashed and soaked in the original liquid for an additional 96 hours. After 5 minutes of centrifugation at 4000 r/min, the saturated solution was added separately to one liter of petroleum ether, trichloromethane, ethyl acetate, or *n*-butanol. These four distinct types of extracted liquor were collected for lyophilization and stored at −20°C, respectively.

### 2.2. Antibacterial Effect of BRF Extracts

Bacterial strains of *Staphylococcus aureus* (*S. aureus*) ATCC25923 and *Escherichia coli* (*E. coli*) ATCC25922 were obtained from the Pathogenic Microbiology Laboratory of Basic Medicine College of Dali University. The *Streptococcus agalactiae* (*S. agalactiae)* strain ATCC12386 was provided by Guangdong CGMCC (Guangzhou, China). These strains were inoculated in a suitable medium using the agar-streak method. When a large independent colony formed in the culture dish, 1-2 colonies were harvested and diluted to 107 cfu/mL in saline solution. The three types of bacteria were then mixed in equal proportions and used within 15 minutes. Two hundred microliters of the BRF extract diluted at concentrations of 10.00, 5.00, 2.50, 1.25, 0.63, 0.31, 0.16, 0.08, 0.04, and 0.02 mg/mL were added into 96-well plates simultaneously with 20 *μ*L of bacterial suspension and 220 *μ*L of the medium. The minimum inhibitory concentration (MIC) and minimum bactericidal concentration (MBC) were measured by the broth microdilution method. MIC is defined as the lowest concentration of an antimicrobial that will inhibit the visible growth of a microorganism after overnight incubation, and MBC is the lowest concentration of the antimicrobial that will prevent the growth of an organism after subculture onto antibiotic-free media, while the minimum concentration of MIC wells that contained CFU ≤5 was MBC.

For the preparation of cream at concentrations of 100 mg/g, 200 mg/g, and 300 mg/g, the extracts with the lowest MIC and MBC were vigorously mixed with a cream base, which is primarily composed of stearic acid, Vaseline, liquid paraffin, glycerin, and triethanolamine.

### 2.3. Animal Model

Forty-eight female SD rats (SPF, 200–220 g, aged 12+ weeks) were purchased from Hunan Slack Laboratory Animal Co., Ltd. (Changsha, China), with vaginal cleanness of I-II and improved Donders score ≤2. Estradiol benzoate (Ningbo second pharmaceutical industry, Ningbo, Zhejiang, China) was subcutaneously injected at 0.05 mL/100 g qid thrice for pretreatment. The AV model was established by infusing 0.5 mL of bacterial suspension into the vagina qid on four occasions. For treatment, AV rats were grouped into 6 groups as follows: normal group (group A, without any treatment); paired group (group B, cream base), clindamycin group (group C, clindamycin phosphate vaginal cream at 20 mg/g) (Conba Pharmaceutical Co., Ltd., Hangzhou, Zhejiang, China), low-concentration extract group (group D, extracts at 100 mg/g), medium-concentration extract group (group E, extracts at 200 mg/g), and high-concentration extract group (group F, extracts at 300 mg/g). Rats were treated with the corresponding cream by vaginal smearing qid for 14 consecutive days. At the end of the treatment, all rats were weighed and sacrificed, and vaginal samples were collected. We used swabs for vaginal sampling, similar to the human leucorrhoea collection, and separated vaginal tissue for 10% formaldehyde fixation and freezing at −80 degrees.

### 2.4. Vaginal Secretion pH and Improved Donders Score Analysis

Rat vaginal secretion pH was measured by pH test paper (3.8–7.0). The improved Donders score was calculated using five indices, including *Lactobacillus* grading-LBG, white blood cell count, background colonies, peripheral blood cells' percentage, and submucosal hyperemia. The total score was divided into the following categories: 0–2: no AV, 3-4: mild AV, 5-6: medium AV, and 7–10: severe AV.

### 2.5. Hematoxylin and Eosin (HE) Staining for Histopathologic Evaluation

Following the sacrifice of the rats, vaginal tissue was sectioned and fixed in 10% neutral formalin, embedded in paraffin blocks using a microtome (Leica RM2255, Germany), and stained for histological analysis. In brief, samples immersed in xylene and alcohol were stained with hematoxylin for 5 minutes, then with eosin for 3 minutes, followed by being reimmersed in alcohol and xylene. Slides were then mounted using a synthetic resin (Entellan; Merck, Germany) and graded on a scale from 1 (most superficial) to 60 (deepest) using Olympus CX23 (Tokyo, Japan).

We commissioned two pathologists from the affiliated hospital of Dali University to evaluate inflammatory pathological changes. Because the Shapiro–Wilk test showed that the data of each group did not meet a normal distribution, the measurement data were expressed as the median (interquartile spacing), namely, *M* (*Q*). Histopathological scoring criteria for vaginal inflammation in rats include mucosal lesion (the squamous epithelium was edema, thickened, structural disorder, cell necrosis, and epithelial keratosis) 0–3, submucosal hyperemia 0–3, and inflammatory cell infiltration 0–3.

### 2.6. Immunohistochemistry Staining

The H&E staining of formalin-fixed, paraffin-embedded (FFPE) tissues was previewed. Sections suitable for immunohistochemistry were deparaffinized in xylene, rehydrated in a series of graded alcohols, and autoclaved for five minutes in ethylenediaminetetraacetic acid (EDTA). Endogenous peroxidase was blocked with 3% H_2_O_2_. Tissue sections were then incubated with a primary antibody against Beclin1 (1 : 200) or LC3B (1 : 200) (Beclin1 mouse polyclonal antibodies and LC3B mouse polyclonal antibodies (Sanying Biotechnology Co., Ltd., Wuhan, China)) overnight at 4°C. Images were captured by Olympus DP26 (Tokyo, Japan), and average optical density (AOD) was measured using Image-Pro Plus 6.0 (Media Cybernetics Image Technology Inc., Maryland, USA).

### 2.7. Western Blot

Isolated proteins (30 mg) were separated by electrophoresis on a 15% sodium dodecyl sulfate (SDS) gel and then transferred onto a polyvinylidene difluoride (PVDF) membrane. The membrane was blocked for 3 hours in Tris-buffered saline containing 0.1% Tween 20 (TBST; 50 mM Tris-Cl, pH 7.5, and 150 mM NaCl) and 3% bovine serum albumin (BSA) and subsequently incubated overnight at 4°C with a primary antibody against Beclin1(1 : 2000) or LC3B (1 : 2000). The membrane was then washed thrice with TBST and incubated with an appropriate horseradish peroxidase-conjugated secondary antibody (1 : 2000) (Beyotime Biotechnology Co., Ltd., Shanghai, China). Finally, the protein bands detected by antibodies were visualized using enhanced chemiluminescence (ECL) reagent (Thermo Fisher Scientific, Waltham, MA, USA).

### 2.8. Enzyme-Linked Immunosorbent Assay (ELISA)

Beclin1 and total LC3B proteins in serum were detected using ELISA kits (Sunred Biotechnology Company, Shanghai, China), according to the manufacturer's instructions. Serum Beclin1 and total LC3B protein levels were calculated by measuring absorbance at a wavelength of 450 nm using microplate reader 1681133A (Bio-Rad Laboratories Inc., California, USA).

### 2.9. Statistical Analysis

SPSS Statistics for Windows, version 17.0 (Chicago: SPSS Inc.), was used for statistical analysis. Comparisons between the data before and after the treatment were performed using the paired *t*-test. The data were analyzed by one-way ANOVA to determine the variance between multiple groups and then by LSD to compare two groups. A Pearson correlation test was used to analyze the correlation between Beclin1 and LC3B protein expression. *P* < 0.05 represented statistical significance.

## 3. Results

### 3.1. The *In Vitro* Antibacterial Effect of Four BRF Extracts

Antibacterial effects of BRF extracts were evaluated by the microdilution broth method in 96 wells. As shown in Table 1, the petroleum ether and *n*-butanol extracts of BRF had no antibacterial activity at the maximum concentration of 10.00 mg/mL. In contrast, the ethyl acetate extract exhibited the most potent antimicrobial effects and bactericidal activity with the lowest MIC and MBC, suggesting it the best candidate for the subsequent *in vivo* studies.

### 3.2. Effect of the Ethyl Acetate Extract on Vaginal Secretion pH and Improved Donders Score

After establishing the AV model in rats, Donders scores improved, reaching diagnostic levels of severe AV. Following treatment, scores in the paired and low-concentration extract groups remained unchanged. In contrast, scores decreased in the clindamycin, medium-concentration extract, and high-concentration extract groups. Additionally, the medium-concentration extract group scored higher than the clindamycin group, which scored higher than the high-concentration extract group.

**Figure 1:**
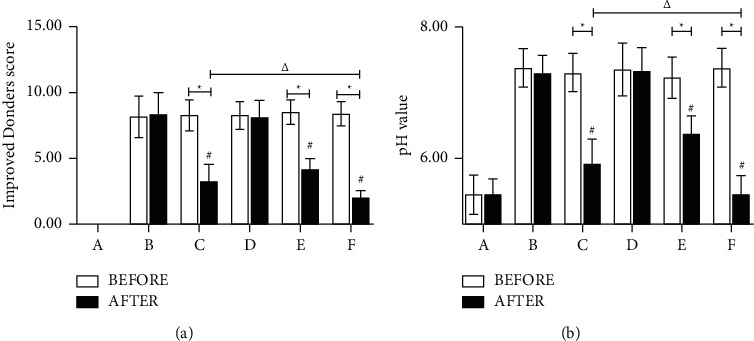
Effect of ethyl acetate extracts on vaginal secretion pH and improved Donders score. After treatment, the value of group A (normal group), group B (paired group), group C (clindamycin group, 20 mg/g), group D (low-concentration extract group, 100 mg/g), group E (medium-concentration extract group, 200 mg/g), and group F (high-concentration extract group, 300 mg/g). (a) Vaginal secretion improved Donders score. (b) Vaginal secretion pH value. Note: ^*∗*^comparison before and after the same drug treatment, *P* *<* 0.05; ^#^compared with the paired group, *P* *<* 0.05; ^△^compared with the clindamycin group, *P* *<* 0.05.

pH of vaginal secretion increased after duplicating the model. After treatment, there was no change in the paired and low-concentration extract groups, but there was a decrease in the clindamycin, medium-concentration extract, and high-concentration extract groups. Additionally, pH was higher in the medium-concentration extract group than in the clindamycin group, higher than in the high-concentration extract group (Figures 1(a) and 1(b)).

### 3.3. Effect of the Ethyl Acetate Extract on H&E Staining and Histopathology Score

**Figure 2:**
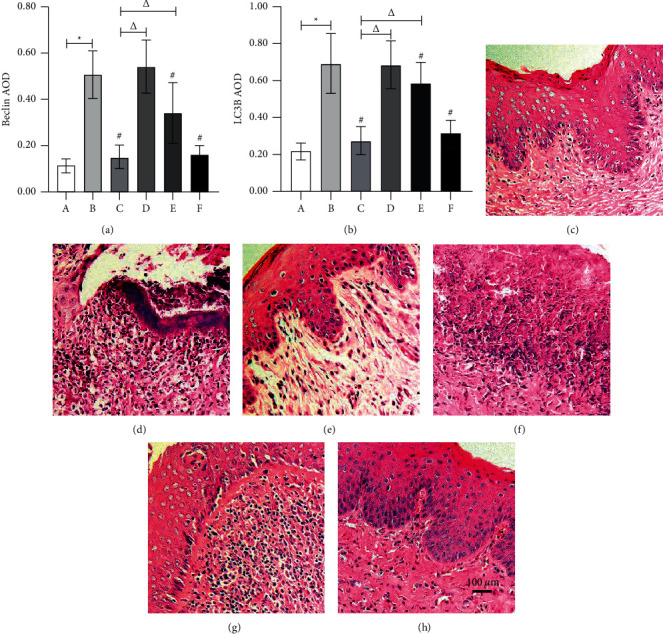
AOD values of Beclin1 and LC3B and H&E staining (×100) in the AV model. AOD means average optical density. (a) AOD value of Beclin1 in vaginal tissue. (b) AOD value of LC3B in vaginal tissue. (c) Group A (normal group). (d) Group B (paired group). (e) Group C (clindamycin group, 20 mg/g). (f) Group D (low-concentration extract group, 100 mg/g). (g) Group E (medium-concentration extract group, 200 mg/g). (h) Group F (high-concentration extract group, 300 mg/g).

A significantly abnormal higher histopathology score was observed in AV rats. As shown in Table 2 and Figures 2(c)–2(h)), low-concentration extract had no effect. In contrast, medium- and high-concentration extracts significantly ameliorated mucosal lesions, with a dose-dependent improvement in submucosal hyperemia and inflammatory cell infiltration. The AOD of Beclin1 and LC3 was significantly higher in the paired group than in the normal group, remained unchanged in the low-concentration extract group compared to the clindamycin group, while decreased significantly in the clindamycin group, medium-concentration extract group, and high-concentration extract group compared to the paired group. The inhibitory effect of the extract on Beclin1 and LC3 in groups E and F is dose-dependent.

### 3.4. Effect of the Ethyl Acetate Extract on Beclin1 and LC3B Expressions

**Figure 3:**
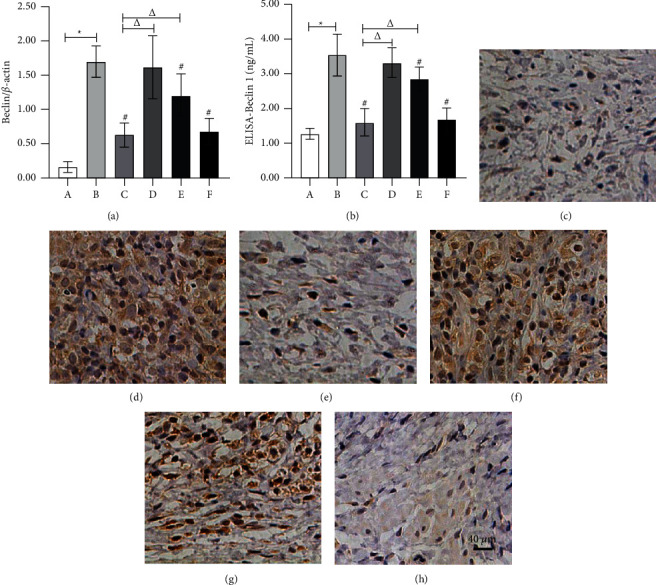
Beclin1 expression in each group. (a) Beclin1/*β*-actin by western blot. (b) Beclin1 serum concentration by ELISA. Beclin1 ICH (×400): (c) group A (normal group), (d) group B (paired group), (e) group C (clindamycin group, 20 mg/g), (f) group D (low-concentration extract group, 100 mg/g), (g) group E (medium-concentration extract group, 200 mg/g), and (h) group F (high-concentration extract group, 300 mg/g). Note: ^*∗*^compared with the normal group, *P* < 0.05; ^#^compared with the paired group, *P* < 0.05; ^△^compared with the clindamycin group, *P* < 0.05.

**Figure 4:**
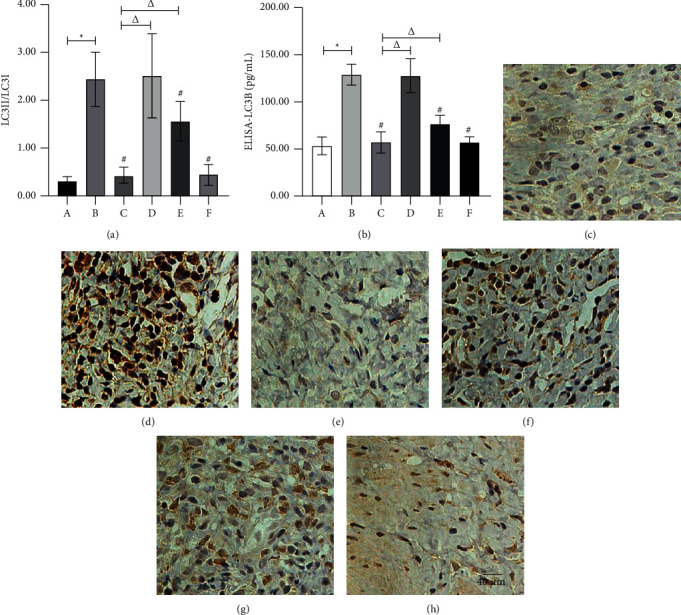
LC3B expression in each group. (a) The detection of LC3B-II/LC3B-I by western blot. (b) The LC3B serum concentration detected by ELISA. (c–h) The LC3B ICH (×400) of group A (normal group) (c), group B (paired group) (d), group C (clindamycin group, 20 mg/g) (e), group D (low-concentration extract group, 100 mg/g) (f), group E (medium-concentration extract group, 200 mg/g) (g), and group F (high-concentration extract group, 300 mg/g) (h). Note: ^*∗*^compared with the normal group, *P* < 0.05; ^#^compared with the paired group, *P* < 0.05; ^△^compared with the clindamycin group, *P* < 0.05.

**Figure 5:**
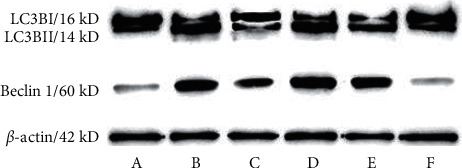
Beclin1 and LC3B proteins detected by western blot. Group A (normal group), group B (paired group), group C (clindamycin group, 20 mg/g), group D (low-concentration extract group, 100 mg/g), group E (medium-concentration extract group, 200 mg/g), and group F (high-concentration extract group, 300 mg/g).

The Beclin1 protein was stable in the cytoplasm and nuclear envelope but only weakly expressed in normal vaginal tissue. In contrast, it was expressed in the AV model's entire epithelial layer, subcutaneous, pericapillary, and part mesenchyme. Beclin1 protein expression decreased after treatment (Table 3, Figures 3(a)–3(h), and Figure 5). In normal vaginal tissue, LC3B protein was expressed stably but weakly in the stratified squamous epithelium and subcutaneous tissue surrounding capillaries. However, in the AV model, LC3B protein was expressed in all epithelial and subcutaneous layers, and its expression decreased following treatment (Table 3, Figures 4(a)–4(h), and Figure 5). The elevated Beclin1 and LC3B protein levels in the AV model remained unchanged in the low-concentration extract group. However, in the clindamycin, medium-concentration extract, and high-concentration extract groups, they were significantly downregulated, with the dose-dependent effects being equivalent in the high-concentration extract and clindamycin groups but weaker in the medium-concentration extract group.

On the contrary, the Beclin1 and LC3B's serum concentrations remained lower in the physiological state. At the same time, they increased significantly in the AV model. No change was observed in the low-concentration extract group, but in the clindamycin, medium-concentration extract, and high-concentration extract groups, there was a significant decrease. The treatment was equally effective in the clindamycin and high-concentration extract groups but less effective in the medium-concentration extract group.

### 3.5. Correlation between Beclin1 and LC3B in the AV Model

Immunohistochemical, western blot, and ELISA data of Beclin1 and LC3B had a normal distribution. Statistical analysis revealed *P* > 0.05 and correlation coefficient *r* (Table 4), indicating that Beclin1 and LC3B have no linear dependence on the physiological status, AV status, and antibiotic or BRF extract treatment (Table 4).

## 4. Discussion

AV has a high incidence and recurrence rate, but antibiotics tend to develop resistance, and new medication is urgently needed. BRF is widely distributed in Yunnan, Bai, and Yi ethnics having a long history of use and recording. When stimulated, the insects would secrete an irritating dark brown defense liquid that would prove to have antibacterial activity [8]. In the general population, the insects have been used to treat fever, injury, and tumor by drying, grinding, or marinating in liquor.

The current study of BRF was focused on classification, component identification, and pharmacomechanism [3, 9, 10], including antioxidant, antineoplastic, and anti-inflammatory activities. For the antibacterial activity, it had been verified that BRF extracts could inhibit and eliminate multiple bacteria and fungi, including *S. aureus*, *E. coli*, *S. agalactiae*, *Bacillus subtilis, Pseudomonas aeruginosa,* and *Candida albicans*, but only *in vitro* and not in animal models. In this study, we used the method of 60% ethanol extraction to explore the *in vitro* antibacterial activity of different polar parts of BRF extracts on standard AV pathogens, including *S. aureus*, *E. coli,* and *S. agalactiae*. Our results showed that MID and MBD of the ethyl acetate extract were the lowest, consistent with previous studies [11–13], suggesting that ethyl acetate extracts could be the best antibacterial candidates to treat AV in the rat model. We also found that medium- and high-concentration BRF ethyl acetate extracts were as efficacious as clindamycin in AV rats. After 14 days of vaginal administration, rats' vaginal orifice redness dissipated, secreta reduced, Donders score improved, pH of vaginal secretion improved, and histopathology score decreased in varying degrees. These results indicate that medium- and high-concentration BRF ethyl acetate extracts have a therapeutic effect, and the dosage of BRF may have clinical applications in the future and can ameliorate symptoms of AV inflammation.

Autophagy has a double-edged sword effect on infections and pathogens, invasion-induced xenophagy, and lysosomal-dependent degradation [14]. Molecules involved in autophagy regulation in infectious disease include property pattern recognition receptor (PRR), autophagy receptor sequestosome 1/p62-like receptor (SLR), and ubiquitin-like modified protein (UBL) autophagy-related (ATG) proteins. SLR interacted with LC3B to recognize ubiquitin and initiate autophagy, forming an autophagosome that carried LC3B and parceled pathogen before being processed to the lysosome for degradation [15]. Autophagy remained low in normal rats, implying that probiotics do not need to upregulate autophagy in vaginal mucosa when confronted with inflammatory response and pathogen invasion in the physiological state. Vaginal epithelial cells (VECs) with active autophagy survived the damage caused by *C. albicans* invasion, indicating that autophagy plays a critical role in the survival of human VECs during infection [16].

Infection with *S. aureus, E. coli,* or *S. agalactiae* can activate autophagy in VECs, is not limited to degradation, but instead “hijacks” autophagy, and accelerates proliferation to induce autophagy-dependent host cell death. In our AV rat model, immunohistochemistry showed granules with intense positive staining in Beclin1, and LC3B, with AOD, scored higher than in the control group. Beclin1 and LC3B expression levels were higher in the AV model than in the control by western blot and ELISA. Three of the above bacteria induced excessively activated autophagy, which suppressed the protective effect of probiotic-related moderately upregulated autophagy, and high-level autophagy, which participate in AV pathogen invasion and proliferation.

The larva, imago, and defense liquid of BRF, a pharmaceutical insect, have strong antibacterial activities. Luo et al. reported that the liposoluble constituent of the BRF ethyl acetate extract has *in vitro* bacteriostatic activity against *E. coli, Bacillus subtilis, Streptococcus A*, and *Bacillus megaterium* [17]. Shi et al. demonstrated that the BRF ethyl acetate extract has better bacteriostatic activity against several common bacteria, especially *S. aureus* (MID: 3.13 mg/mL) and *Staphylococcus albus* (MID: 1.30 mg/mL) [12, 13]. Li et al. discovered that the BRF alcohol extract could alleviate the inflammatory symptoms of aseptic granuloma in mice and subcutaneous and aseptic prostatitis in rats [5, 18]. Numerous studies demonstrate that BRF extracts have promising therapeutic effects on both infectious and noninfectious inflammation. However, the molecular mechanisms underlying these effects remain unknown.

## 5. Conclusions

In this study, we found that, in the AV rat model, clindamycin and BRF ethyl acetate extracts at a medium or high concentration have comparable therapeutic effects on infection and autophagy. Immunohistochemistry revealed that clindamycin weakened Beclin1 and LC3B granules, while AOD score, western blot, and ELISA detected that clindamycin decreased Beclin1 and LC3B expressions. The inhibitory effect of medium- and high-concentration BRF ethyl acetate extracts on Beclin1 and LC3B expressions was dose-dependent, implying that antibiotics and extracts exerted their therapeutic effects by inhibiting autophagy. Our previous research found that antioxidants can decompose reactive oxygen species (ROS) and may lead to the inhibition of autophagy in the endometriosis rat model [19]. The preliminary research on the therapeutic effect of the BRF ethyl acetate extract on AV demonstrated that the extract achieved its objective by inhibiting excessive activated autophagy of infective vaginal tissue. Following this, we will conduct an in-depth study of the extract's effect with a proper concentration on autophagy key gene regulation, bacterial and autophagy subcellular localization, and other fields of application for insect-derived drugs.

## Figures and Tables

**Table 1:** The antibacterial effect of four BFR extracts on three bacteria *in vitro* (mg/mL).

Bacterium	Petroleum ether		Trichloromethane		Ethyl acetate		*n*-Butanol	
	MIC	MBC	MIC	MBC	MIC	MBC	MIC	MBC
*E. coli*	—	—	2.50	2.50	1.25	1.25	—	—
*S. aureus*	5.00	5.00	1.25	5.00	0.63	1.25	5.00	10.00
*S. agalactiae*	2.50	5.00	1.25	1.25	0.08	0.31	10.00	10.00

**Table 2:** Histopathology score of vaginal tissue in each rat group *M* (*Q*) (*n* = 8).

Group	Concentration (mg/kg)	Histopathology score			
		Mucosal lesions	Submucosal hyperemia	Inflammatory cell infiltration	Total score
Normal	0	0 (0.75)	0 (0.75)	0.50 (1.00)	1.00 (0)
Paired	0	3.00 (0.75)^a^	3.00 (0)^a^	3.00 (0)^a^	8.50 (1.00)^a^
Clindamycin	20	0.50 (1.00)^b^	1.00 (1.00)^b^	0 (0.75)^b^	1.50 (1.00)^b^
Low	100	3.00 (0.75)^c^	3.00 (1.00)^c^	3.00 (0)^c^	8.00 (0.75)^c^
Medium	200	2.00 (1.00)^bc^	1.50 (1.00)^bc^	2.00 (0)^bc^	5.00 (0.75)^bc^
High	300	0 (1.00)^b^	0 (1.00)^bc^	1.00 (0.75)^bc^	2.00 (1.50)^bc^

*M* (*Q*): median (interquartile range); ^a^compared with the normal group, *P* < 0.05; ^b^compared with the paired group, *P* < 0.05; ^c^compared with the clindamycin group, *P* < 0.05.

**Table 3:** Grey level of Beclin1 and LC3B by western blot ($\bar{x}\pm s$, *n* = 8).

Group	Dosage (mg/kg)	Beclin1/*β*-actin	LC3B-II/LC3-BI
Normal	—	0.16 ± 0.08	0.29 ± 0.11
Paired	—	1.70 ± 0.23^a^	2.44 ± 0.56^a^
Clindamycin	20	0.63 ± 0.18^b^	0.43 ± 0.17^b^
Low	100	1.62 ± 0.46^c^	2.51 ± 0.87^c^
Medium	200	1.21 ± 0.31^bc^	1.56 ± 0.41^bc^
High	300	0.68 ± 0.19^b^	0.43 ± 0.22^b^

^a^Compared with the normal group, *P* < 0.05; ^b^compared with the paired group, *P* < 0.05; ^c^compared with the clindamycin group, *P* < 0.05.

**Table 4:** Pearson's correlation analysis of Beclin1 and LC3B.

Group	Concentration (mg/kg)	Immunohistochemical		Western blot		ELISA	
		*r*	*P*	*r*	*P*	*r*	*P*
Normal	0	0.578	0.113	0.457	0.089	0.463	0.248
Paired	0	0.493	0.214	0.612	0.136	0.682	0.063
Clindamycin	20	0.658	0.076	0.429	0.915	0.648	0.082
Low	100	0.339	0.412	0.603	0.257	0.662	0.074
Medium	200	0.634	0.092	0.377	0.302	0.732	0.039
High	300	0.614	0.105	0.482	0.091	0.685	0.061

## Data Availability

The data used to support the findings of this study are available from the corresponding author upon request.
